# Editorial: Tumor microenvironment and metabolic reprogramming in cancer

**DOI:** 10.3389/fimmu.2024.1497966

**Published:** 2024-10-08

**Authors:** Xiaoling Cui, Xinyu Liu, Rongjie Feng, Xiaohan Wang, Yiju Wei, Houjuan Zhu, Asif Raza, Xingyu Zhu, Hao Chen, Wei Chong

**Affiliations:** ^1^ Department of Gastrointestinal Surgery, Shandong Provincial Hospital Affiliated to Shandong First Medical University, Jinan, China; ^2^ Shandong Provincial Laboratory of Translational Medicine Engineering for Digestive Tumors, Shandong Provincial Hospital, Jinan, China; ^3^ Medical Science and Technology Innovation Center, Shandong First Medical University & Shandong Academy of Medical Sciences, Jinan, China; ^4^ Department of Orthopedics, Shandong Provincial Hospital Affiliated to Shandong First Medical University, Jinan, Shandong, China; ^5^ School of Life Science, Shandong First Medical University & Shandong Academy of Medical Sciences, Jinan, Shandong, China; ^6^ Institute of Materials Research and Engineering (IMRE), Agency for Science, Technology and Research (ASTAR), Singapore, Singapore; ^7^ Department of Pharmacology, Penn State Cancer Institute, The Pennsylvania State University College of Medicine, Hershey, PA, United States; ^8^ Clinical Research Center of Shandong University, Clinical Epidemiology Unit, Qilu Hospital of Shandong University, Jinan, China

**Keywords:** cancer, metabolic reprogramming, TME (tumor microenvironment), tumor, multi omics

The tumor microenvironment (TME) is a complex and dynamic entity that plays a crucial role in cancer progression, treatment resistance, and metastasis (1). One of the most significant aspects of the TME is the metabolic reprogramming that occurs in both cancer cells and the surrounding stromal and immune cells (2). The following editorial synthesizes insights from eleven recent studies that highlight the intricate relationship between the TME, metabolic pathways, and cancer development, offering new avenues for therapeutic intervention.

A recent review by Long et al. explores how tumor metabolism affects immune cells in the TME. Metabolic byproducts like lactate and reactive oxygen species can impair the function of immune cells, creating an immunosuppressive environment that shields the tumor from immune attack. Understanding these interactions is key to developing strategies to restore immune function and enhance immunotherapy efficacy. In addition, the role of specific metabolic pathways in cancer progression is researched by Zhou et al. The identification of key genes related to tryptophan metabolism, such as NAMPT, IDO1, and ACAT1, as significant biomarkers in BLCA, points to the potential of these pathways as targets for therapy. Similarly, Li et al. find that using machine learning to identify genes related to glutamine metabolism in breast cancer patients provides a new approach for prognostic modeling, which has the potential to guide more effective and less toxic personalized treatment strategies.

A recent review by Li et al. emphasizes the importance of glycolysis, often upregulated in cancer cells to meet energy demands. Key enzymes like hexokinase, phosphofructokinase, and pyruvate kinase are highlighted for their role in cancer progression, along with related signaling pathways and transcription factors as potential therapeutic targets. The review suggests targeting glycolysis could provide new therapeutic strategies for improving ovarian cancer treatment and prognosis.

The TME is also influenced by hormonal and inflammatory factors, which can significantly impact cancer progression (3). A recent review by Wenxuan et al. discusses how steroid hormones like androgens, estrogens, and progestins influence colorectal cancer growth and therapy response, suggesting targets for hormone-based treatments. Additionally, Habanjar et al. highlights that the pro-inflammatory environment in obese patients can impair the protective role of myoepithelial cells, promoting cancer progression, and underscores the importance of considering obesity in breast cancer treatment and prognosis.

Peng et al. identify circ_BBS9 as a potential biomarker and therapeutic target in lung adenocarcinoma (LUAD), noting its downregulation in LUAD tissues and association with poor prognosis. Circ_BBS9 interacts with IFIT3, influencing immune infiltration in the tumor microenvironment. Similarly, Fan et al. find that increased CD8+ T cell infiltration is linked to poorer prognosis in prostate cancer, suggesting that targeting MLXIPL, related to these T cells, could improve outcomes, particularly in immunotherapy.

The influence of the TME on cancer progression is further explored in the context of different cancer types. For instance, Kim et al. studies the aryl hydrocarbon receptor (AhR) in various solid tumors and reveales its differential expression in cancer cells and immune cells within the TME. These findings suggest that AhR could serve as a therapeutic target, particularly in cancers where its expression is associated with poor prognosis.

Moreover, the emerging role of extracellular vesicles (EVs) in the metastatic process, particularly in brain metastases (4), underscores the complexity of the TME. The recent research by Li et al. highlights the potential of EVs as biomarkers for early detection and as targets for therapeutic intervention, particularly in enhancing the delivery and efficacy of drugs targeting brain metastases.

As research advances, natural agents like epigallocatechin gallate (EGCG) show promise for more effective and less toxic cancer therapies. Li et al.‘s review highlights how EGCG impacts the tumor microenvironment, metabolism, and immunotherapy efficacy, enhancing immune responses and suppressing immunosuppressive cells, making it a potential boost for cancer immunotherapy and treatment outcomes.

In conclusion, the studies discussed in this editorial provide a comprehensive overview of the dynamic interplay between tumor metabolism and the microenvironment, highlighting the potential of targeting these interactions to improve cancer treatment. As our understanding of these processes deepens, it opens the door to more effective, personalized therapies that not only target the cancer cells but also modulate the TME to enhance immune responses and overcome therapeutic resistance. The future of cancer therapy lies in the integration of metabolic and immunological insights, paving the way for innovative approaches that could transform patient outcomes.
